# Cost-utility analysis of cardiovascular outpatient rehabilitation care and spa treatment care for patients with heart disease

**DOI:** 10.1186/s12962-020-00236-6

**Published:** 2020-09-29

**Authors:** Ondřej Gajdoš, Vojtěch Kamenský, Kristýna Doskočilová, Martina Caithamlová, Ivana Kubátová

**Affiliations:** Department of Biomedical Technology, Faculty of Biomedical Engineering, Czech Technical University in Prague, nám. Sítná 3105, 272 01 Kladno, Czech Republic

**Keywords:** Cardiovascular rehabilitation, Outpatient treatment, Spa treatment, Health related quality of life, Cost-utility analysis

## Abstract

**Background:**

Cardiovascular diseases have the highest mortality rates and the costs for treatment are very high so far. Cardiovascular rehabilitation helps to reduce the risk of relapses or deterioration of cardiovascular diseases, however, the number of patients that participate is insufficient, especially in later stages of the rehabilitation process. The aim of the study is to evaluate cost-effectiveness of cardiovascular rehabilitation care using cost-utility analysis.

**Methods:**

The study evaluate the Cardio ambulance Late Phase, Late Phase of The Spa treatment and for comparison also Early Phase of The Spa treatment in Konstantin Spa. The research was conducted in outpatient facility and spa facility. For QALY, a prospective questionnaire survey was conducted in patients with cardiovascular disease using generic EQ-5D-5L questionnaires. The costs were calculated from the perspective of the health care payer. The cost-utility analysis was carried out at the end of the study and results are presented in incremental cost-utility ratio.

**Results:**

The average cost per patient in outpatient facility is CZK 12,459. The average amount for an overall early phase of spa treatment per patient is CZK 35,161. The average amount for an overall late phase spa treatment per patient is CZK 30,503. QALY obtained from Index Value was 0.092 (Konstantin Spa Early Phase), 0.054 (Konstantin Spa Late Phase), 0.26 (Cardio ambulance Late Phase). For Konstantin Spa Late Phase, the ICUR value was 644,436 and for Konstantin Spa Early Phase was 343,981 (comparator is the Cardio ambulance Late Phase). Konstantin Spa Early Phase compared to Konstantin Spa Late Phase had an ICUR value of 122,592.

**Conclusions:**

The results of this study suggest that the spa treatment in later stage of the cardiovascular rehabilitation process is cost effective with use of cost effectiveness threshold three times the Gross Domestic Product (GDP) per capita.

## Background

Due to the ever-evolving technology in health care, the quality and life expectancy of the population are increasing. This phenomenon also applies to the treatment of cardiovascular diseases which are the main cause of mortality in the Czech Republic and in Europe [1]. In addition to the proven effectiveness of pharmacological treatment, the treatment of non-pharmacological, above all invasive cardiac surgery, has been largely used thanks to which mortality in the acute stage of cardiovascular disease has decreased in recent years. However, the patient is not cured by the surgery and care should be taken to eliminate the risk of a disease progression. Such an option is non-pharmacological cardiovascular rehabilitation. Although there is evidence of its clinical effect the participation in this type of treatment is inadequate as well as the awareness of its potential.

Cardiovascular rehabilitation (CV RHB) is a comprehensive approach that helps to keep optimal physical, mental, social, professional and emotional state. It doesn’t only include physical activity but also monitors compliance with the principles of secondary prevention and lifestyle changes. The European Cardiology Society and the Czech Society of Cardiology are both seeking to increase awareness among cardiologists as well as the general public about the rehabilitation of patients with cardiovascular diseases. At the same time, these institutions are working on the implementation of professionally handled rehabilitation as a necessary part of the treatment [2, 3].

The goal of the rehabilitation care is to motivate the patient to a lifelong physical activity and an overall lifestyle change. The main condition is to participate in a positive change in morbidity and mortality by thoroughly monitoring compliance with the principles of secondary prevention and endeavouring to positively influence risk factors. Physical rehabilitation care is the principle element in this complex [2].

The form of cardiovascular rehabilitation depends on the nature and equipment of a cardiology clinic. Group exercises with the cooperation of a cardiologist or possibly also psychologist and physiotherapist are proven to be valuable. In this case, physical rehabilitation is suitably supplemented by psychological instruction about proper dietary habits. Equally as effective may also be individual exercise check-ups [2].

Spa therapy has a positive effect lasting for several months. It has a positive influence on the patient’s personality and its psychological effect is also indisputable, e.g. in geriatric patients, which is again reflected into their physical and mental health. The role of spa is significant and difficult to substitute in the treatment of chronic illnesses and rehabilitation after severe acute illnesses, operations and injuries [4]. Cardiac rehabilitation treatment is specific in each European country. This specificity for the Czech and Slovak Republics, and partly also for Germany, has always been the use of natural healing resources [5].

In recent years in the concept of cardiac rehabilitation, there has been a shift away from classical, predominantly passive, procedures. Although these procedures were perceived as very positive by patients, they treated primarily associated illnesses. In the modern concept of spa cardiac rehabilitation treatment, emphasis is placed on an active approach. The patient is under the physician’s supervision motivated to comply with dietary guidelines, supervised exercise, and suitable lifestyle [6].

Spa treatment of patients with cardiovascular disease should follow after hospitalization (or outpatient rehabilitation) in cases of acute illness or invasive treatment, and especially in case of chronic conditions. It leads to a faster improvement of physical and psychological health as well as establishment and consolidation of healthy lifestyle habits, elimination of risk factors and suppression of the impacts of psychological stress [2].

The aim of this study is to compare two specific types of cardiovascular rehabilitation: outpatient treatment against spa treatment in selected healthcare facilities using cost-utility analysis (CUA).

## Methods

The research was conducted in two different types of health care facilities. Kardioambulance, s.r.o. in Prague 9—Prosek was chosen as an outpatient facility (Cardio ambulance). The facility, as one of the few in the field of diagnosis and treatment, provides outpatient cardiovascular rehabilitation services, offers standard rehabilitation care and specialized care for patients with cardiovascular disease. As a spa facility we reached out to Konstantin Spa, a.s., which focuses on spa treatment of patients with cardiovascular disease and locomotor system disease.

**Fig. 1:**
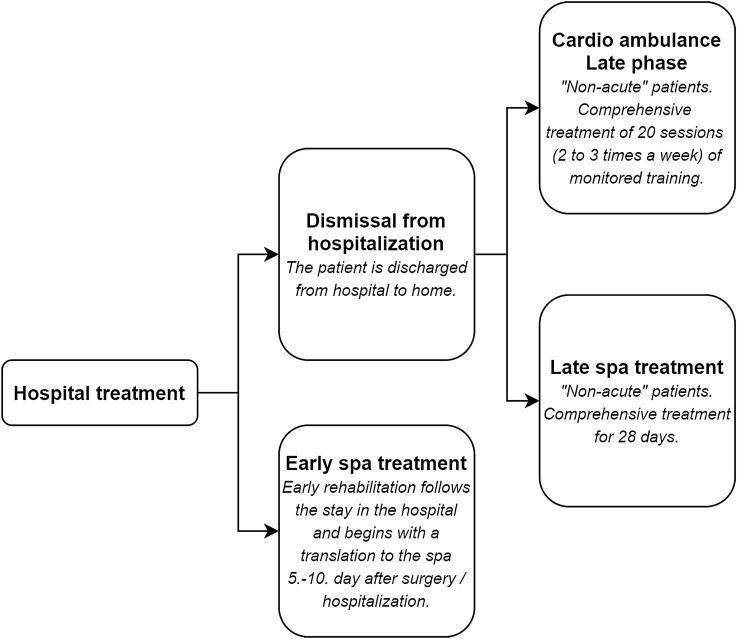
Division of patients into treatment groups

With patients transported directly from the hospital, the physical and mental state at the beginning of treatment is very unstable but improves rapidly during treatment. With patients transported from their home, the physical and mental condition is in most cases stabilized and improves gradually. The group of patients transported from their home is comparable to patients who undergo outpatient treatment. In terms of CV RHB phases, transported spa patients from the hospital are at an early stage of rehabilitation, while spa patients from home and outpatient patients are in a late rehabilitation phase. For the purposes of this study, it is necessary to compare these two groups of spa treatments separately, both from the perspective of obtained Quality-adjusted life year (QALY) and the subsequent elaboration of the CUA. The division of patients into groups is shown in the Fig. 1.

The cost-utility analysis was calculated from the perspective of the health care payer, i.e. the health insurance companies in the Czech Republic. Incremental cost-utility ratio (ICUR) was used for evaluation. The one-way sensitivity analysis was calculated as the sensitivity analysis. Due to the low number of probands and lack of further information, it was decided to analyse the sensitivity to the given parameters in the range of 30%. There was also a scenario in which the duration of effect is the same for Cardio ambulance treatment and Late Spa treatment.

### QALY

For obtaining QALY, a prospective questionnaire survey was conducted in patients with cardiovascular disease using generic EQ-5D-5L questionnaires, which is a standardised instrument for use as a measure of health outcome developed by the EuroQol group [7]. Information on the duration of the treatment effect has been obtained from the physicians of the healthcare facilities. In the outpatient facility, a questionnaire survey ran from November 30, 2015 to March 31, 2016, with a total of 63 questionnaires completed before and after the treatment. Data collection from 7th March to 15th April 2016 took place at the spa facility with a total of 88 questionnaires completed before and after the treatment.

Patient selection was based on the following criteria:

- Patients with cardiovascular disease,
- Comprehensive treatment for 28 days in patients at the spa,
- Comprehensive treatment of 20 sessions (2 to 3 times a week) of monitored training in patients in outpatient settings.

Index values before and after treatment were evaluated using the EuroQol EQ-5D-5L conversion calculator and were averaged for each group. Questionnaire analysis was conducted using German preferences, because there isn’t standard values set for Czech Republic. A prerequisite for choosing German preferences is that they are neighboring countries Europe and both are high-income countries. The physicians (cardiologists) of the given healthcare facility determined the duration of the effect for each type of treatment. These values were converted to QALY units.

### Costs

When estimating the costs from the perspective of the health care payer, i.e. health insurance companies, information from the contracts of the insurance companies with the given healthcare facilities, the reimbursement ordinance, the code list and data provided by the facility employees were used.

The reimbursement of health care in a given outpatient facility is determined by a combination of payments for performance and a flat-rate payment over a given period. The health insurance companies regulate the specific conditions, in particular the value of the points for performance and the amount of the flat-rate.

The reimbursement of the health care in the given spa is carried out in accordance with Decree No. 273/2015 Coll., for 1 day at the spa. This amount is made up of a payment for housing, meals and treatment. The amount of the reimbursement is determined by the contractual relationship between the health insurance company and the healthcare institution for each particular group of circulatory system disease.

## Results

### QALY

A data summary from the EQ-5D-5L questionnaires for individual groups of patients in the spa facility and outpatient patients is shown in Tables 1 and 2. The most significant improvement (Index Value difference) occurred in patients in the early stage of CV RHB, followed by patients from outpatient facility and on the third place are spa treatment patients in late phase of CV RHB. The effect of duration was determined based on a discussion of a group of 6 independent experts composed of cardiologists, rehabilitation physicians and physiotherapists. After recalculation to QALY values, due to the short duration of the effect in outpatient patients, this group moved to third place in the value of the QALY obtained, while the first one is still occupied by early RHB patients, and the second place is hold by the spa patients of the late RHB. The overview of the questionnaire survey evaluation based on the index value is shown in Table 1.

**Table 1:** Overview of the questionnaire survey evaluation (index value)

	Konstantin Spa early phase	Konstantin Spa late phase	Cardio ambulance late phase
Probands number	58	30	63
Index value before treatment	0.710	0.761	0.747
Index value after treatment	0.833	0.833	0.852
Index value difference	0.123	0.072	0.105
Effect duration	9 months	9 months	3 months
QALY obtained index value	0.092	0.054	0.026

**Table 2:** Overview of outpatient facilities outputs and their value for the health insurance companies

Performance	Points	Number of performance	Point value^a^ [CZK]	Amount^a^ [CZK]	Point value^b^ [CZK]	Amount^b^ [CZK]
Therapeutic physical education (TPE) individual under supervision on machines	81	40	0.8	2592.00	0.8	2592.00
TPE individual-fitness and analytical methods	78	40	0.8	2496.00	0.8	2496.00
Telemetric electrocardiographic (ECG) monitoring outpatient settings	414	10	1.03	4264.20	1.04	4305.60
Targeted examination performed by a cardiologist	354	1	1.03	364.62	1.04	368.16
ECG examination performed by a specialist	141	1	1.03	145.23	1.04	146.64
Highly specialized echocardiographic examination	1064	1	1.03	1095.92	1.04	1106.56
Specialized ergometric examination	609	2	1.03	1254.54	1.04	1266.72
Screening (Orientation Spirometry)	37	4	1.03	152.44	1.04	153.92
Pulse Oximetry	77	1	1.03	79.31	1.04	80.08

^a^ General Health Insurance Company (VZP), Czech Industrial Health Insurance Company (ČPZP), Military Health Insurance of the Czech Republic (VoZP), Health Insurance Company of the Ministry of the Interior of the Czech Republic (ZPMV) [8–16]

^b^ Branch Health Insurance Company (OZP) [15–17]

### Cost estimation in outpatient facilities

Comprehensive treatment of CV RHB includes various types of performance that are reported to outpatient patients by health insurance companies. An overview of these outputs reported per patient in a given outpatient setting with their values is given in Table 2.

General Health Insurance Company (VZP), Czech Industrial Health Insurance Company (ČPZP), Military Health Insurance of the Czech Republic (VoZP), Health Insurance Company of the Ministry of the Interior of the Czech Republic (ZPMV) spend CZK 12,444 for the treatment of one patient while Branch Health Insurance Company (OZP) insurance company spends CZK 12,516. The cost per patient was taken from the overall number of patients insured with the given health insurance company in the provided sample. The average cost per patient is CZK 12,459. In total, the health insurance companies spent CZK 784,917 on the given sample of patients.

### Cost estimation in spa facilities

The reimbursement is determined by the contractual relationship between the health insurance company and the healthcare facility for each particular indication group. Health insurance companies generated costs for 1 day of spa treatment at a given spa facility are given in Table 3.

**Table 3:** Overview of the cost per one day of spa treatment [12, 18–21]

Indication number	VZP	ZPMV	VoZP	ČPZP
II/1	**–**	**–**	**–**	921.50
II/2	1041	1058	1057.50	1053.50
II/3	1009	1022	1021.50	1021.50
II/4	1009	1022	1021.50	1021.50
II/5	1018	1041	1021.50	1030.50
II/6	1045	1058	1317.50	1317.50
II/7	–	–	–	957.50
II/8	1045	1058	1057.50	1057.50
II/9	1045	1058	1317.50	1057.50
II/99 (II/6)	1305	–	–	–
Average	1065	1046	1116	1065

Indicator group II/99 covers all conditions after cardiac surgery as defined in original indication II/6, involving a direct transfer from an acute healthcare facility. VZP spends most on Group II/99 treatment, with an average amount of CZK 1065 for a treatment a day. ZPMV spends its highest amount (CZK 1058) on Indicator Groups II/2, II/6, II/8 and II/9, with an average cost per treatment per day being CZK 1044.79. The insurance company VoZP directs its highest amount (CZK 1317.50) towards Indicator Groups II/6 and II/9, with an average cost per treatment per day being CZK 1116. ČPZP pays the highest amount for a treatment per day (CZK 1317.50) for the indicator group II/6. The average amount per treatment per day is 1065 CZK. OZP has a contractual relationship with the given spa facility, which is governed by Section 15 of Decree No. 273/2015 Coll., i.e. CZK 1050 per day, with the maximum contractual amount settled for 2016 being CZK 2500,000 [15, 22].

Tables 4 and 5 show the cost conversion per patient according to the number of patients insured with the given health insurance company in the given sample who participated in research at the spa facility.

**Table 4:** Health insurance company’s costs per patient—early phase of spa treatment [15, 18–23]

Indicator group	Insurance company	Amount per day	Number of days	Number of patients	Total amount
II/6	VZP	1305.00	28	35	1,278,900
	ZPMV	1057.50	28	9	266,490
	OZP	1050.00	28	3	88,200
	ČPZP	1317.50	28	5	184,450
	VoZP	1317.50	28	6	221,340
Total				58	2,039,380

**Table 5:** Health insurance company’s costs per patient—late phase of spa treatment [15, 18–23]

Indicator group	Insurance company	Amount per day	Number of days	Number of patients	Total amount
II/2	VZP	1041.00	28	2	58,296
	ZPMV	1058.00	28	2	59,248
	OZP	1050.00	28	0	0
	ČPZP	1053.50	28	0	0
	VoZP	1057.50	28	0	0
II/3	VZP	1009.00	28	1	28,252
	ZPMV	022.00	28	2	57,232
	OZP	1050.00	28	0	0
	ČPZP	1021.50	28	1	28,602
	VoZP	1021.50	28	0	0
II/6	VZP	1045.00	28	12	351,120
	ZPMV	1058.00	28	4	118,496
	OZP	1050.00	28	1	29,400
	ČPZP	1317.50	28	2	73,780
	VoZP	1317.50	28	3	110,670
Total				30	915,096

The health insurance companies spent CZK 2039,380 on the treatment of 58 early-transferred patients. The average amount for an overall treatment per patient is CZK 35,162.

Health insurance companies spent 915 096 CZK on a late phase of spa treatment of 30 patients. The average amount for an overall treatment per patient is CZK 30,503.

### Cost-utility analysis

The cost-utility analysis was calculated from the health care payer perspective, i.e. the health insurance companies. ICUR was calculated from the available results. The ICUR is defined as the ratio of the cost difference of a particular treatment intervention to the difference in their utilities. It expresses the amount of cost per unit of extra utility, i.e. the price for the extra QALY obtained. The cheaper variant (Cardio ambulance Late Phase) was chosen as the comparator and ICUR1 was calculated in comparison especially with the late phase and then also with early phase of the spa treatment in Konstantin Spa. After that Spa Late Phase was chosen as the comparator and ICUR2 was calculated with Spa Early Phase. In general, the value of three times the Gross Domestic Product (GDP) per capita is taken as the limit of efficiency in the world [24]. According to the Czech Statistical Office, the value of GDP per capita for 2015 is CZK 435,911 [25]. Three times this value is CZK 1307,733. The ICUR results are shown in Table 6. Although no ICUR value exceeds the threshold, the strategy Konstantin Spa Late Phase is dominated by strategy Konstantin Spa Early Phase—we can observe the so-called weak dominance (extended dominance). From the point of view of the care process, the Cardio ambulance corresponds more to the strategy Konstantin Spa Late Phase, so in the following sensitivity analyses we do not focus on the strategy Konstantin Spa Early Phase.

**Table 6:**
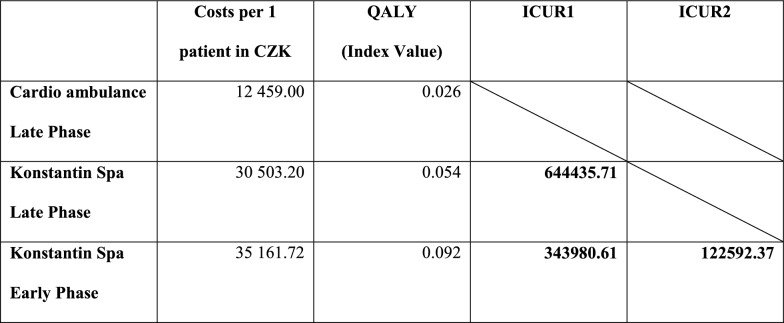
Incremental cost utility ratio (QALY obtained from Index Value)

The results of the one-way sensitivity analysis are shown in Table 7. The table shows the results of the comparison of therapy in Cardio ambulance and Konstantin Spa Late phase.

**Table 7:** One-way sensitivity analysis indicates the ICUR value at 30% parameter change

Parameter	Base case	Lower value	Upper value	ICUR base case	ICUR lower value	ICUR upper value
Index value difference Cardio ambulance	0.105	0.0735	0.1365	644,436	506,504	907,884
Index value difference Konstantin Spa	0.072	0.0504	0.0936	644,436	1562,268	410,562
Effect duration Cardio ambulance	3	2	4	644,436	494,362	949,695
Effect duration Konstantin Spa	9	6	12	644,436	1850,687	394,409
Average cost Cardio ambulance	12,459	8721.3	16,196.7	644,436	784,933	515,550
Average cost Konstantin Spa	30,503.2	21,352.2	39,654.2	644,436	320,477	980,006

The parameters with the greatest effect on the ICUR result are the Konstantin Spa Index value difference and the Effect duration Konstantin Spa reduced by 30%. In these cases, the ICUR is above the willingness to pay (WTP) and therefore the Late spa intervention is not cost effective. Index value difference Cardio ambulance, Effect duration Cardio ambulance and Average cost Konstantin Spa parameters increased by 30%, had an effect on ICUR, but the value did not exceed the WTP level in these cases. Because of the assumptions of the effect duration, scenario analysis was performed. The results of scenario with the same effect duration are shown in Table 8.

**Table 8:**
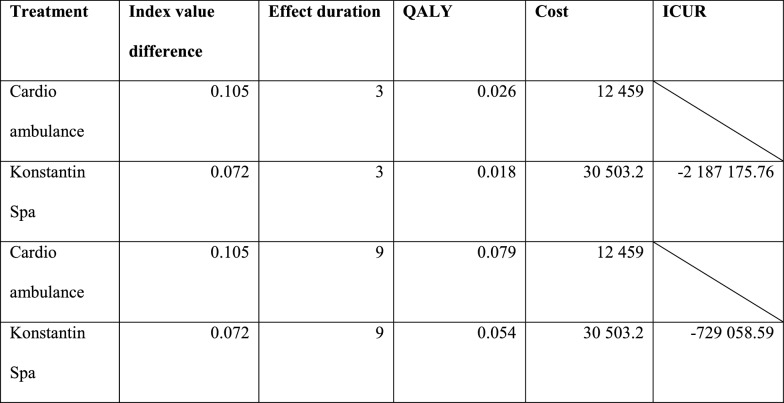
Results of scenario with the same effect duration

Scenarios with the same duration of effect have an effect on the ICUR of the two compared treatment approaches in late stage cardiovascular rehabilitation. In this case, Cardio ambulance care is dominant over spa treatment. Furthermore, in the sensitivity analysis was calculated how long the late phase of spa effect must last in order to late phase of spa intervention remain cost-effective. The duration of the effect of late phase of spa must be more than 4.375 months.

## Discussion

Cardiovascular diseases are the number one cause of mortality in most industrially developed countries. In Europe, about 4 million people die annually from cardiovascular disease. In a group of people under 65, cardiovascular disease has a 31% mortality rate for men and 26% for women. For the southern states, particularly France and Spain, long-term low cardiovascular mortality rates are typical. This fact is associated with the so-called nutritional paradox (French paradox), which is a contradiction between not so healthy eating and low cardiovascular mortality. It is believed that this phenomenon is related to the positive influence of the Mediterranean diet and lifestyle. In the Czech Republic, cardiovascular disease contributed to total mortality with 43% in 2014 [1].

Treatment of people with cardiovascular disease is divided into pharmacological and non-pharmacological. Non-pharmacological treatment includes invasive procedures and cardiovascular rehabilitation. Despite the great merit of acute surgery, due to modern technology and advances in medicine consideration should be given to preventing recurrence or progression of the disease. This is where cardiovascular rehabilitation and physical activity come to the picture. It is a very effective and when following the guidelines also a safe tool to positively affect the risk factors such as overweight, hypertension, dyslipidaemia or insulin resistance. Unfortunately, CV RHB and physical activity are still largely under-represented among the Czech population [26]. Physical activity is an integral part of cardiovascular rehabilitation. Into this area, we also include education about healthy lifestyle and the associated elimination of possible risk factors, especially smoking cessation programs, nutritional counselling and psychological counselling. A Canadian study dealing with the state of cardiovascular rehabilitation services conducted a research in 40 countries where CV RHB services were provided in 26 of them, and in most of them, the rehabilitation program consisted of physical exercise, nutrition counselling, psychological counselling and anti-smoking programs [27].

In the Czech Republic, a spa treatment which is well-accessible from a demographic point of view is still a great tradition. However, from 2010 to 2016, there was a significant decrease in the number of patients, by 64%. In 2016, therefore, a total of 4885 adults with a circulatory disorder received a spa treatment at the cost of a health insurance company. According to the published statistics [28], this trend can be caused by both a change in the health care reimbursements system from the side of the health insurers and an attempt to return to working life as soon as possible.

Another option for performing cardiovascular rehabilitation is outpatient treatment. There aren’t many facilities that provide this type of service comparing to spas. The reason may be, among other things, costly and complex devices equipment that is required to operate such service, the already mentioned need for comprehensive care including movement component, dietary measures and psychological counselling and the necessary training of health professionals in order to provide specialized rehabilitation care [29].

The difference in the perception of these two types of cardiac rehabilitation is in a sense quite substantial and it needs to be mentioned. The spa treatment of the circulatory system as well as the spa treatment in general is perceived by the patient as a specific one-time type of recreation where the patient finds himself in a different environment outside the working process, surrounded by people with similar difficulties, and his stay has a regimen of clearly defined, scheduled procedures. This kind of treatment usually has a very positive impact on the patient and the treatment effect can last for several months. After completing the treatment and returning to a “normal” life, however, it often happens that people slowly return back into their old habits they used to have before the treatment and their lifestyle is once again moving towards a negative direction. For cardiac patients, this can mean a return to poor dietary habits, physical inactivity or stress. And these factors, despite the patient’s motivation to continue a healthy lifestyle even outside the spa, can lead to a worsening of the disease. Outpatient rehabilitation treatment is perceived by the patient as a necessity to “go somewhere” twice a week. During the treatments, the patient finds himself in his common professional and personal circumstances and in a relatively short time it should encourage him or her to actively cooperate and change his/her lifestyle. It is not an unusual phenomenon that a patient who is at first apathetic and inactive is experiencing much more enthusiasm after a few weeks of exercise than during the first visit. Therefore, the patient is encouraged during ambulatory and spa cardio rehabilitation to make this treatment not just a one-time matter but a part of his lifestyle. As found from the study, the most significant difference between these two types of treatments were reflected in the duration of positive effect after the treatment determined by the cardiologists of the facilities. While the more intense spa treatment was rated with a lasting effect of 9 months, the duration of the effect of outpatient treatment after attending 20 sessions was 3 months. It should be emphasized that ambulatory CV RHB achieves the greatest effects after 2 years of treatment. A meta-analysis of 63 randomized trials involving a total of 21,295 patients was performed by Clark AM et al. [30]. The study shows that the 12-month CV RHB reduces the recurrence of myocardial infarction by 17%. After 2 years of rehabilitation, mortality is reduced by 47%.

In the questionnaire survey framework, the aim was to carry out a research in patients (cardiac patients) without focusing on a specific diagnosis. This decision was made because of how time-consuming data collection is and in order to get more respondents in case of one diagnosis. The suitability of a selected sample of patients was consulted with a cardiologist. The programs of both therapeutic interventions were identical for all patients in the groups. The groups were divided according to CV RHB phase into a group of patients undergoing early spa rehabilitation treatment and two groups of patients undergoing spa and outpatient rehabilitation care at a later stage of CV RHB. Groups equal and thus relevantly comparable are the last two groups mentioned, as evidenced by similar values of the Index value (0.726 and 0.747) of the EQ-5D-5L questionnaires at the start of treatment. A group of early rehabilitation patients are mentally and physically unstable, and in the questionnaire survey, the Index value is significantly lower (0.710) before treatment. Therefore, it cannot be considered as an equivalent comparator to other groups, but due to the existence of data, it was ranked and processed for comparison. The results of the questionnaire survey show that at a later stage, CV RHB achieved greater improvement, i.e. a greater difference in the Index value of outpatient treatment patients. The resulting QALY value is higher for spa patients due to the higher duration of the effect.

Effect values, especially effects of late spa treatment, most influenced the ICUR value in the sensitivity analysis. In particular, Konstantin Spa Index value difference and the Effect duration Konstantin Spa had a significant impact on ICUR and decision about cost-effectiveness of the treatment. In another words, effect of late spa treatment must be 1.5 times longer than effect of Cardio ambulance.

Based on the results of the study, recommendations for cardiovascular rehabilitation can be established. The cardiac rehabilitation of cardiac patients at the early stage of CV RHB had the greatest clinical effect. Therefore, we can cautiously say that this type of treatment has its place in cardiovascular rehabilitation and, based on the experience of the spa, there is a marked improvement in the physical and mental health of the patient. To confirm cost effectiveness, we would need to create another study that would have a suitable comparator which the other two groups did not meet in this study. The results of the main two groups of patients at the later stage of CV RHB have shown better cost-effectiveness ration (C/U) in outpatient cardiovascular rehabilitation. Outpatient treatment is therefore a suitable alternative to spa treatment at a given stage of rehabilitation, especially in productive patients, in terms of saving the cost of lost earnings from employment. Due to the increasing prevalence of chronic forms of cardiovascular disease, the late rehabilitation phase is very relevant. However, it is not only necessary to increase the availability of given rehabilitation care but also to improve the awareness of the possibilities of cardiovascular rehabilitation, not only among patients but also among professionals (physicians). These were one of the reasons why we decided to carry out this study [31].

## Conclusions

The study compares two therapeutic interventions using Health Technology Assessment (HTA) methods. The aim of the work was to analyse the costs and benefits and to compare the given types of treatment based on its results. These therapeutic interventions were compared on the basis of the HTA methodology. A cost-utility analysis has been developed based on the treatment outcomes. Costs were processed from the perspective of the health care payer. Benefits were evaluated based on obtained QALY (using generic EQ-5D-5L questionnaire). The results were processed and concluded that outpatient treatment seems to be more cost effective in patients with late stage cardio rehabilitation (in terms of cost per QALY). The late phase of spa option could be considered cost-effective but at more than double the costs, it may not be an affordable alternative to outpatient treatment (Cardio ambulance Late Phase).

## Data Availability

The datasets used and/or analysed during the current study are available from the corresponding author on reasonable request.

## Abbreviations

ČPZP: Czech Industrial Health Insurance Company
CV RHB: Cardiovascular rehabilitation
ECG: Electrocardiograph(ic)
GDP: Gross Domestic Product
HTA: Health Technology Assessment
ICUR: Incremental cost effectiveness ratio
OZP: Branch Health Insurance Company
QALY: Quality-adjusted life year
TPE: Therapeutic physical education
VAS: Visual analogue scale
VoZP: Military Health Insurance of the Czech Republic
VZP: General Health Insurance Company
WTP: Willingness to pay
ZPMV: Health Insurance Company of the Ministry of the Interior of the Czech Republic
